# Study Engagement and Burnout of the PhD Candidates in Medicine: A Person-Centered Approach

**DOI:** 10.3389/fpsyg.2021.727746

**Published:** 2021-11-23

**Authors:** Lotta Tikkanen, Kirsi Pyhältö, Aleksandra Bujacz, Juha Nieminen

**Affiliations:** ^1^Centre for University Teaching and Learning, Faculty of Educational Sciences, University of Helsinki, Helsinki, Finland; ^2^School of Applied Educational Science and Teacher Education, Philosophical Faculty, University of Eastern Finland, Joensuu, Finland; ^3^Faculty of Education, University of Oulu, Oulu, Finland; ^4^Behavioral Informatics Team, Health Informatics Centre, Department of Learning, Informatics, Management, and Ethics, Karolinska Institutet, Stockholm, Sweden; ^5^Department of Learning, Informatics, Management, and Ethics, Karolinska Institutet, Stockholm, Sweden

**Keywords:** burnout, drop-out, PhD candidate, research engagement, well-being

## Abstract

This study focused on exploring individual variations in doctoral candidates’ well-being, in terms of experienced research engagement and burnout by using a person-centered approach. In addition, the associations between well-being profiles and gender, country of origin, study status (full-time or part-time), research group status and drop-out intentions were explored. The participants were 692 PhD candidates in the field of medicine. Latent profile analysis was employed to identify the well-being profiles. Four distinct profiles were identified: *high engagement–low burnout, high engagement–moderate burnout, moderate engagement–moderate burnout*, and *moderate engagement–high burnout.* Working in a clinical unit or hospital and working in a research group seemed to be related to increased engagement and reduced risk for suffering burnout, while the intentions to quit one’s doctoral studies were more frequently reported in profiles with moderate levels of engagement. The findings imply that although a significant number of PhD candidates in medicine had an increased risk for developing burnout, for most of the PhD candidates research education is an engaging experience.

## Introduction

Undertaking a doctoral degree provides both highs and lows, potentially significantly reducing or increasing PhD candidates’ well-being (e.g., Stubb et al., 2011; Divaris et al., 2012; Caesens et al., 2014; Hunter and Devine, 2016; Swords and Ellis, 2017). Yet, previous research on the topic has focused heavily on the negative attributes such as stress (e.g., Oswalt and Riddock, 2007; Pappa et al., 2020), depression (e.g. Peluso et al., 2011; Levecque et al., 2017), anxiety (e.g., Barry et al., 2018; Liu et al., 2019), and exhaustion (e.g., Hunter and Devine, 2016), while positive aspects of PhD experience have been studied to a lesser extent (Barnes and Randall, 2012; Sverdlik et al., 2018; Pyhältö et al., 2019). In particular, the number of studies exploring the combination or co-existence of positive and negative attributes of PhD candidates’ well-being is limited (for an exception, see Stubb et al., 2011), although PhD candidate’s well-being cannot be reduced simply to an absence of negative experiences (Schmidt and Hansson, 2018).

A large body of research has indicated that the risk of burnout among physicians and other health care workers is high (van Vendeloo et al., 2018; Dyrbye et al., 2020; Woo et al., 2020). The COVID-19-pandemic has further increased the risk of burnout among health care workers (Chirico et al., 2021; Magnavita et al., 2021). In contrast, we know little about the well-being of research-active employees in the medical fields. Based on the literature on doctoral education, PhD candidates working in the medical context have rarely been studied. The medical research context is affected by the culture and hierarchy of the wider organizational culture of health care and hospital hierarchy, likely affecting PhD candidates’ well-being (Kusurkar et al., 2021). Furthermore, there are at least two distinct subgroups of PhD students in these contexts (Naylor et al., 2016): those who also work clinically and those working in the basic sciences. These two groups of PhD candidates often work under very different conditions, within the same medical university setting (Naylor et al., 2016). More context-specific studies into PhD candidates in medical research education and the differing subgroups of PhD candidates in medicine have been called for (Naylor et al., 2016; Kusurkar et al., 2021).

In this study, we aimed to explore the individual variation in well-being among PhD candidates in medicine by employing a person-centered approach. We focused on identifying burnout-engagement profiles employed by PhD candidates in the medical fields, and how they are related to working in a clinical unit or hospital, study status (full-time or part-time), research group status, and drop-out intentions. Also, differences between international and native (Swedish) PhDs candidates, and men and women were examined.

### PhD Candidates’ Well-Being

PhD candidates’ study well-being is a multidimensional construct referring to a combination of positive mental states, such as satisfaction, self-efficacy or/and study engagement, and absence of extensive and severe negative ones such as burnout or strain related to doctoral studies, further contributing to a candidates ability to pursue their study goals (Korhonen et al., 2014; Widlund et al., 2018). Study well-being is constructed in an interplay between demands and resources of the PhD. candidate and their doctoral study environment (see on study well-being among undergraduates Salmela-Aro and Upadyaya, 2014). In this study, we explore PhD. candidates’ study well-being in terms of study engagement and burnout. It has been suggested that s*tudy engagement* is a symbol of an optimal PhD experience, characterized by vigor, dedication, and absorption (Schaufeli et al., 2002b; Salmela-Aro and Upadyaya, 2012). Among PhD candidates, engagement is typically manifested as high levels of energy and mental resilience while working with one’s doctoral research, a strong willingness to invest effort in the doctorate, a sense of significance, enthusiasm, and inspiration, and being fully focused on one’s work, whereby time passes quickly (Virtanen and Pyhältö, 2012; Vekkaila et al., 2013, 2014). Engagement in doctoral study has been shown to be positively related to study progress and negatively to drop-out intentions (Castelló et al., 2016).

*Study burnout*, in turn, refers to a negative study experience that is characterized by two core symptoms, exhaustion and cynicism, resulting from prolonged stress (Schaufeli et al., 2002a; Salmela-Aro et al., 2009). Exhaustion refers to lack of emotional energy and chronic fatigue (Maslach and Jackson, 1981), and cynicism refers to alienation from one’s studying, perceiving them as meaningless and losing interest in them (Maslach, 2003). Burnout during doctoral study has been shown to be related to delaying doctoral study and intending to quit them (Pyhältö et al., 2012; Anttila et al., 2015; Hunter and Devine, 2016; Cornér et al., 2017; Barry et al., 2018).

In variable-based studies, study engagement and burnout have typically been found to be negatively related to each other (Schaufeli et al., 2002a; González-Romá et al., 2006; Salmela-Aro and Upadyaya, 2012; Swords and Ellis, 2017). This means that the PhD candidates experiencing high levels of study engagement are likely to experience low levels of study burnout and vice versa. However, various combinations of study engagement and burnout are also possible (Tuominen-Soini and Salmela-Aro, 2014; Salmela-Aro and Read, 2017). For example, a PhD candidate can be highly engaged in their doctorate, but simultaneously experience high levels of exhaustion. A reason for this might the gradual development of burnout: burnout typically begins with exhaustion, and then, if working conditions remain the same, also the levels of cynicism increase (Maslach and Leiter, 2016). Studies using a person-centered approach to explore PhD candidates’ study engagement and burnout simultaneously are scarce, resulting in a lack of knowledge about individual variations in the study well-being of PhD candidates in medicine. In addition, it is not known how different study well-being profiles are related to individual and contextual factors.

### Antecedents of PhD Students’ Study Well-Being

Research has identified several individual and contextual antecedents of PhD candidates’ well-being. For instance, gender has been shown to be associated with study well-being, yet the evidence is mixed: although there is some evidence showing that female PhD students experience more stress and exhaustion than males (Toews et al., 1997; McAlpine et al., 2020), there is also evidence of male postgraduates being more likely to experience increased levels of exhaustion than their female colleagues. Hunter and Devine (2016), on the other hand, showed that PhD students’ gender was not associated with their experiences of exhaustion. The mixed findings imply that gendered impact may be dependent on the socio-cultural or disciplinary practices.

Some differences between international and native PhD candidates have also been reported. It has also been suggested that international PhD candidates are more career-oriented and more satisfied with their doctoral studies, which might make them more likely to experience research engagement compared to native PhD candidates (Harman, 2003; Sakurai et al., 2017). However, international PhD candidates have also been shown to experience stress due to a lack of a supportive network (Pappa et al., 2020), which increases their risk of burnout. Yet, evidence concerning the differences between domestic and international PhD candidates’ well-being is particularly limited.

Working conditions can be expected to have an impact on the well-being of PhD candidates in the medical fields. First, it has been suggested that the PhD candidates who are involved in clinical work experience high work strain due to constant balancing with their clinical or patient responsibilities and PhD research (Kusurkar et al., 2021), which makes them prone to burnout experiences. On the other hand, there is also evidence that real work-life experiences such as clinical work can inspire candidates in their doctoral studies, and thus contribute to increased engagement (see Vekkaila et al., 2013). In a qualitative case study, comparing clinically active and basic science PhD candidates in the same context, Naylor and others (2016) showed that clinical doctoral candidates were initially less competent in basic research skills than candidates who had learned these skills at earlier stages of their basic science education. An adjustment from an established position at the clinic to being a junior researcher in the laboratory was challenging. On the other hand, financial stress characterized the experience of the science candidates more than that of the clinicians. Clinical PhD candidates also saw research education as being more clearly connected to career opportunities in the future than their basic science counterparts in the same setting did. Perceived employment opportunities have been associated with lower burnout levels in biomedical PhD candidates (Nagy et al., 2019). Differences in the working conditions of medical PhD candidates may thus affect the levels of burnout and engagement in differing ways.

Research group status, i.e., whether the PhD candidate is undertaking their doctoral research within a research group or alone, can be assumed to have impact on study well-being. Research group has been shown to be an important source of social support to PhD candidates, and hence, working in a research group can be assumed to increase the experienced engagement (Stubb et al., 2011; Peltonen et al., 2017). However, it has also been found that working within a research group can be a source of stress (Stubb et al., 2011). Moreover, study status, i.e., whether the PhD candidate is undertaking their degree part-time vs. full-time, may have an impact on their study well-being. Yet, the evidence in this regard is partly contradictory. While those who work full-time are shown to be more satisfied with their supervision and perceive the scholarly community as empowering compared to those who work part-time (Stubb et al., 2011; Pyhältö et al., 2016), candidates working part-time are shown to be more satisfied with their mental health and friendships (Isohätälä et al., 2017).

## Aim of the Study

The aim of the study was to understand the individual differences in study well-being among PhD candidates in medicine. More specifically, we explored the PhD candidates’ study engagement–burnout profiles and their associations with background variables that have previously shown to be associated with PhD candidates’ well-being [i.e., gender, country of origin, and study status (i.e., whether they were completing their doctorate full-time or part-time], and research group status). We also explored whether PhD candidates classified into different study well-being profiles differed in their intensions to drop out from doctoral studies. The following general hypotheses were formulated:

*H1*: Different study engagement–burnout profiles can be detected among PhD candidates in medicine, ranging from profiles with high levels of burnout and low levels of engagement to profiles with low levels of burnout and high levels of engagement.

*H2*: The PhD candidates in the different study well-being profiles differ from each other in terms of gender, country of origin (i.e., domestic/international), and whether they are completing their doctorate full-time or part-time, and whether they work in hospital/clinical unit or not, and whether they worked with their doctorate alone or as a part of a research group (i.e., research group status).

*H3*: The PhD candidates with different study well-being profiles differ in their intentions to quit the doctoral studying, i.e., the students with high levels of burnout and low level of engagement are more likely to consider dropping out from the doctorate than those with low levels of burnout and high levels of engagement.

## Materials and Methods

### Research Context

This study had a cross-sectional design. The data were collected during 2015–2016 through a web-based survey using a secure platform (Artologik). The survey was conducted in English. All PhD candidates at Karolinska Institutet with an activity rate of more than 10%^1^ received an invitation to participate in the survey. Karolinska Institutet is a research-oriented medical university with more than 2000 PhD candidates enrolled. “Medical” is understood as an umbrella term encompassing a wide array of fields with a connection to medicine: From clinical research to a wide variety of basic research topics in microbiological and life sciences. Several allied health sciences, behavioral and medical social sciences, such as nursing, physiotherapy, occupational therapy, psychology, medical ethics, and management are also represented.

All participants were enrolled in the same university-wide research education program and have the same overall formal requirements for their training, regarding the number of credits required from research education courses, general criteria for quality of research work, and basic structures of supervision and quality control of the research education process. However, within that universal organizational framework there is great variation in terms of the topics investigated, practices of individual research groups and supervisors and departmental structures.

There are clinical and basic science PhD candidates at Karolinska Institutet. The clinical PhD candidates typically work within two organizations: The hospital clinic or another health care organization (the manager or supervisor of the clinical work being the person the clinician reports to) and another one in the research group on the university side (the main doctoral supervisor most often being the candidate’s responsible manager). The basic science PhD candidates only work within one organization, the university, and have their main supervisor in doctoral education.

In Sweden, all PhD candidates are fully financed, meaning that they get a monthly salary. Their salary level depends on a variety of factors, mainly the source of finance (for example, grants from abroad, external competitive research funding, research funding from medical industry, or funding provided by the healthcare system for their employees). Clinical PhD candidates typically have considerably higher salaries than their basic science counterparts.

The context of the current study is similar to many other natural science contexts in that much of the research work is done within a research group, and a collaborative “teamwork research training structure” (Chiang, 2003) is prevalent. However, there is considerable variation in this regard. At least two co-supervisors in addition to a main supervisor is an organizational norm.

### Participants

In total, 2044 PhD candidates were invited and 692 responded to the survey (response rate 34%). PhD candidates were all in the medical fields. Of the participants, 61.3% were females and 36.6% males. The age of the participants ranged from 24 to 88, the mean being 35years. Forty six percent of the participants (*n*=320) were Swedish and 53% (*n*=366) were from another country. Of the participants, 67.2% (*n*=465) reported that they were completing their doctorate full-time and 32.7% part-time. Nearly one-third (32.7%, *n*=226) of the participants were working in a hospital or a clinical unit. The proportion of those working mainly on their own with their doctorate was 54.8% (*n*=379), and 44.4% (*n*=307) of the participants reported that they were working in a research team.

Participants were informed that participation was completely voluntary and that they may withdraw from the study at any time without providing any explanation. They were also informed that all of the data which they provided would be strictly anonymous and treated confidentially, responses to the survey would not be linked to any other personal data and that analyses would be made at the group level. Before completing the survey, participants indicated that they had read and understood the information provided above and whether they agreed to participate in the study. The research was approved by the Swedish Central Ethical Review Board (Ref. No#2015/1626-31/5).

### Measures

The participants completed the cross-country doctoral experience (C-DES) survey (see C-DES manual Pyhältö et al., 2018; Castelló et al., 2018). In this study, we used the following C-DES-scales to study PhD students’ study well-being: (1) *research engagement* (5 items) and (2) burnout in studying consisting of two factors: (a) *exhaustion* (4 items) and (b) *cynicism* (5 items). All items were rated on seven-point scales (1=not at all, 2=very rarely, 3=rarely, 4=sometimes, 5=often, 6=very often, 7=all the time; See Appendix 1 for the items). Mean variables were formed to represent research engagement, exhaustion, and cynicism in studying. The Cronbach alpha reliability and descriptive statistics of the subscales are shown in Table 1.

**Table 1:** Descriptive statistics and correlations of the study variables.

	*N*	*α*	*M*	*SD*	Min/Max	1	2	3
1. Exhaustion	692	0.837	3.65	1.19	1/7	–		
2. Cynicism	692	0.895	3.00	1.35	1/7	0.56^**^	–	
3. Engagement	690	0.918	4.90	1.00	1/7	−0.22^**^	−0.60^**^	–

** *p<0.001.*

### Data Analyses

A latent profile analysis (LPA) was used to identify subgroups of individuals based on their experiences of study engagement and burnout. LPA is a person-centered approach that involves grouping individuals into latent classes based on their observed response patterns on specific variables instead of exploring the relationships between the variables (Berlin et al., 2014). LPA provides statistical criteria for model comparisons in selecting the best-fitting number of latent classes and opportunity to include predictors and outcomes compared to other clustering approaches (e.g., Vermunt and Magidson, 2002; Morin et al., 2018). The analyses were carried out using Mplus version 8.6 and MLR estimator that produces maximum likelihood estimates with standard errors and *χ*^2^test statistics that are robust to non-normality (Muthén and Muthén, 1998–2017). Within-class variances were held constant across classes. We used several statistical criteria to choose the best fitting model: The Akaike (AIC), the Bayesian (BIC), adjusted Bayesian (aBIC) information-based measures of fit, and a Vuong-Lo-Mendell-Rubin (VLMR) and Lo-Mendell-Rubin (aLRT), and bootstrapped (BLRT) likelihood ratio tests (Nylund et al., 2007; Berlin et al., 2014). In addition, the theoretical meaningfulness of the profile solution was emphasized in selecting the number of profiles. The average latent class probabilities and entropy values were used to evaluate the clarity of different profile solutions.

To explore whether the PhD candidates with different study well-being profiles differed from each other in terms of background variables (gender, country of origin, working in clinical unit or hospital, study status (full-time or part-time), research group status), we used auxiliary Mplus command (Muthén and Muthén, 1998–2017). The background variables were included as antecedents of the latent class variable while accounting for the measurement error in classification (Asparouhov and Muthén, 2014). This analysis was carried out with the R3STEP procedure of Mplus that performs a multinomial logistic regression and provides the odds ratios describing the effect of background variables on the likelihood of membership in each of the latent profiles compared to other profiles (McLarnon and O’Neill, 2018). DCAT procedure for Mplus was used for examining whether candidates in different profiles differed from each other in terms of their intentions to quit studying for their doctorate.

## Results

### The Study Well-Being Profiles

**Figure 1:**
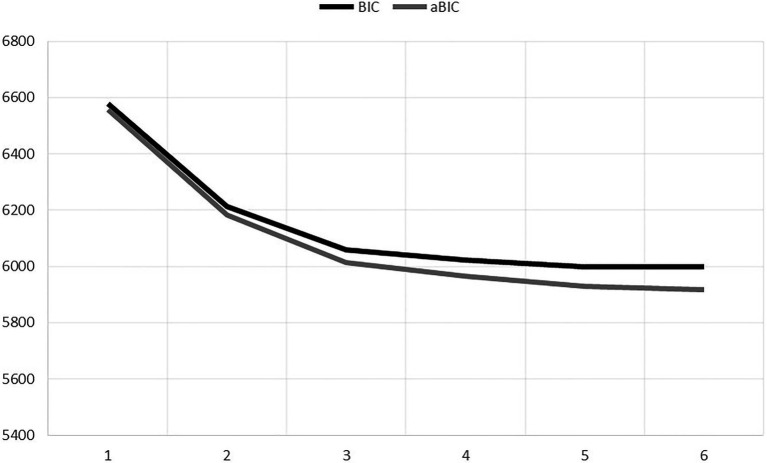
Elbow plot of information criteria for different profile solutions.

LPAs were run with 1–6 classes (Table 2). According to VLMR and aLRT likelihood ratio tests, adding a subsequent class increased the model fit all the way to six classes, while the information criteria (AIC, BIC, and aBIC) showed that adding a new latent profile enhanced the model fit all the way to five profiles. However, the elbow plot (Figure 1) showed that the BIC and aBIC values clearly decreased from one to four profiles, after which the decline levelled off. Therefore, the four-profile solution was selected. The four-profile solution was also considered to be the most parsimonious model, had a clear theoretical interpretation, and included profiles with sufficiently large memberships (i.e., >5% of the cases). The entropy value (0.80) and latent class probabilities (>0.80) also showed sufficient separation between the profiles in the four-profile solution showed sufficient separation between the profiles.

**Table 2:** Information criteria values for different profile solutions in LPAs.

No. classes	LogL (nf)	AIC	BIC	aBIC	Entropy	Latent class probabilities	VLMR	aLRT	BLRT	Class counts^a^
1	−3268.89 (6)	6549.77	6577.01	6557.96	N/A	1.00	N/A	N/A	N/A	
2	−3073.97 (10)	6167.93	6213.33	6181.58	0.72	0.93, 0.89	0.00	0.00	0.00	431, 261 (430, 262)
3	−2983.58 (14)	5995.16	6058.71	6014.26	0.80	0.93, 0.89, 0.90	0.00	0.00	0.00	286, 319, 87 (282, 325, 85)
**4**	**−2953.34 (18)**	**5942.67**	**6024.39**	**5967.23**	**0.80**	**0.93, 0.93, 0.86, 0.87**	**0.01**	**0.02**	**0.00**	**226, 62, 230, 174 (227, 60, 231, 174)**
5	−2927.05 (22)	5898.10	5997.97	5928.12	0.84	0.94, 0.94, 0.90, 0.86, 0.88	0.02	0.02	0.00	222, 7, 68, 222, 173 (223, 7, 69, 224, 169)
6	−2914.54 (26)	5881.09	5999.12	5916.56	0.85	0.92, 0.87, 0.97, 0.93, 0.86, 0.90	0.02	0.02	0.00	9, 170, 7, 221, 222, 62 (7, 169, 7, 223, 223, 63)

*LogL*, *log likelihood value; nf*, *number of free parameters; AIC*, *Akaike information criterion; BIC*, *Bayesian information criterion; aBIC, adjusted Bayesian information criterion; VLMR, VuongLo-Mendell–Rubin likelihood ratio test; aLRT, Lo-Mendell-Rubin adjusted likelihood ratio test; BLRT*, *bootstrapped likelihood ratio test. The selected model is in boldface*.

a *Profile counts based on estimated posterior probabilities and the classification of individuals based on their most likely latent profile membership (in parenthesis)*.

**Figure 2:**
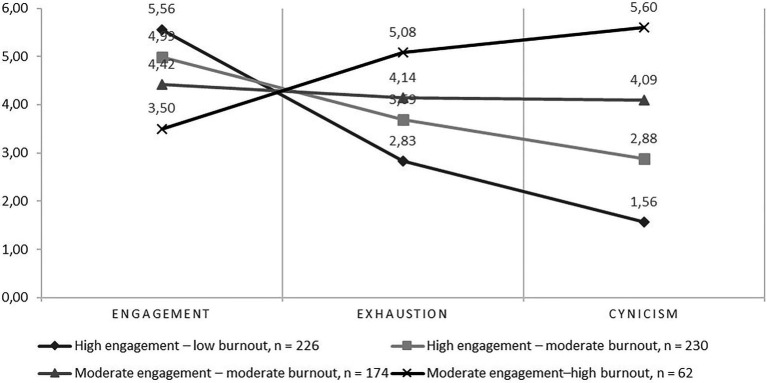
Study well-being profiles of the PhD candidates in medicine.

Four well-being profiles were identified (Figure 2). The first study well-being profile was *high engagement–low burnout* profile (see Table 3). It was the second most common profile among the participants with a 32.7 percent share (*n*=226). The PhD candidates in this profile reported rather high levels of study engagement meaning that they often felt enthusiastic and inspired by their doctoral work. They reported low levels of cynicism, but moderate levels of exhaustion. However, when compared to other profiles, the exhaustion levels were lowest in this profile.

**Table 3:** Profile means and standard deviations.

	*High engagement- low burnout*		*High engagement– moderate burnout*		*Moderate engagement – moderate burnout*		*Moderate engagement–high burnout*	
	M	SD	M	SD	M	SD	M	SD
Engagement	5.56	0.743	4.99	0.709	4.42	0.819	3.50	1.13
Exhaustion	2.83	0.964	3.69	0.932	4.14	0.981	5.08	1.12
Cynicism	1.56	0.437	2.88	0.467	4.09	0.483	5.60	0.578

The second profile was *high engagement–moderate burnout* profile, and it was the most common profile among the participants with a 33.2 percent share (*n*=230). The PhD candidates within this profile reported moderate levels of both exhaustion and cynicism, and high levels of study engagement. The third profile was *moderate engagement–moderate burnout* profile. It represented 25.1 percent of the participants (*n*=174). The PhD candidates with this profile demonstrated moderate levels of study engagement, exhaustion, and cynicism. This means that although the PhD candidates within this profile felt rather inspired and enthusiastic about their doctoral studies, they also sometimes felt overwhelmed by the doctoral study related workload and perceived their doctoral studies as meaningless. The fourth profile was *moderate engagement–high burnout* profile. The PhD candidates with this profile reported high levels of both exhaustion and cynicism. The candidates’ high levels of study burnout were combined with moderate levels of study engagement. This profile represented 9.0 percent of the participants (*n*=62) being the least common profile.

The profiles differed statistically significantly (*p*<0.01) from each other in all study variables, research engagement, exhaustion, and cynicism.

### The Antecedents of Study Well-Being Profiles

Gender and country of origin did not have statistically significant relationships with study well-being profiles. Whether the PhD candidates were completing their doctorate full-time or part-time did not predict the profile membership either.

The PhD candidates who reported that they were working alone with their doctoral thesis had higher odds of belonging to *moderate engagement–high burnout* profile than to *high engagement–moderate burnout* profile (*b*=0.98, SE=0.38, *p*=0.011, OR=2.86, 95%CI[1.25–5.64]) or *high engagement–low burnout* profile (*b*=1.35, SE=0.38, *p*<0.001, OR=4.22, 95%CI[1.83–8.11]) compared to those who were completing their doctorate in a research group. In addition, the PhD candidates who reported that they were working alone with their doctorate had higher odds of belonging to the *moderate engagement–moderate burnout* profile than to the *high engagement—low burnout* profile (*b*=0.83, SE=0.25, *p*=0.001, OR=2.28, 95%CI[1.39–3.75]) compared to those working in research groups.

The PhD candidates who were working in a clinical unit or hospital had higher odds of belonging to *high engagement–low burnout* profile than to *moderate burnout–moderate engagement* (*b*=0.61, SE=0.29, *p*=0.037, OR *=* 1.85, 95%CI[1.04–3.25]) or *moderate engagement–high burnout* (*b*=1.30, SE=0.52, *p*=0.012, OR=3.56, 95%CI[1.30–9.72]) profiles compared to those who reported that they were not working in a clinical unit or hospital. Those working in hospital or clinical unit also had higher odds of belonging to *high engagement–moderate burnout* (*b*=1.23, SE=0.50, *p*=0.015, OR=3.66, 95%CI[1.33–10.10]) profile than to *moderate engagement–high burnout* profile than those who were not working in a clinical unit or hospital.

Taken together, the PhD candidates who reported that they were working alone with their doctorate had higher odds of belonging to profiles displaying lower levels of engagement and higher levels of burnout compared to those working in a research group. In turn, the PhD candidates who reported working in a clinical unit or hospital had higher odds of belonging to profiles displaying higher levels of engagement and lower levels of burnout compared to those who were not at a clinical unit or hospital.

### Differences Between PhD Candidates in Different Profiles in Their Dropout Intentions

The PhD candidates in various profiles differed statistically significantly from each other in terms of their dropout intentions [*χ*^2^ (3, *N*=690)=147.6, *p*<0.001]. The intentions to interrupt one’s doctoral studies were most frequently reported in the following profiles: *moderate engagement–high burnout* profile (74.7%) of the PhD candidates with this profile had considered dropping out) and *moderate engagement–moderate burnout* profile (53.4%). However, the candidates with profiles characterized by high study engagement reported less intentions to interrupt their doctoral studies: 7.2% of the PhD candidates with *high engagement–low burnout* profile and 16.6% with the *high engagement–moderate burnout* profile had considered dropping out.

## Discussion

### Findings in the Light of the Literature

In this study, we explored PhD candidates’ research engagement–burnout profiles. Adopting a person-centered approach allowed us to explore individual variation in PhD candidates’ study well-being by considering both positive and negative attributes of well-being at the same time rather than concentrating on the negative ones which has been the focus of several previous studies (e.g., Oswalt and Riddock, 2007; Peluso et al., 2011; Levecque et al., 2017; Pappa et al., 2020). Four distinct profiles among the PhD candidates in the field of medicine were identified: high engagement–low burnout, high engagement–moderate burnout, moderate engagement–moderate burnout, and moderate engagement–high burnout. The person-oriented approach complements variable-based studies showing a negative association between engagement and burnout (Schaufeli et al., 2002a; González-Romá et al., 2006; Salmela-Aro and Upadyaya, 2012; Swords and Ellis, 2017) by indicating that there are individual differences in how exhaustion, cynicism, and engagement can combine within a person. Our findings supported the bivariant approach on burnout and engagement, positing that burnout and engagement present two distinct, yet related dimensions of the individual’s affective study related experiences (Shraga and Shirom, 2009; Larsen and McGraw, 2011; Shirom, 2011).

The results showed that the levels of research engagement were high or moderate in all the profiles and the most common profiles were those displaying high levels of engagement. Thus, the results indicate that undertaking doctoral studies in the field of medicine is a highly engaging experience. However, the results also showed that the risk of experiencing study burnout was also elevated (i.e., moderate or high) among most of the PhD candidates. These results are in line with earlier findings (Kusurkar et al., 2021) suggesting an increased risk of burnout in medical researcher education.

The results also showed that those PhD candidates who reported working alone with their doctoral studying were more likely to belong to the profiles displaying moderate levels of engagement and higher levels of burnout. This implies that engaging in researcher group provides a potential resource for cultivating not only study progress but also the candidate’s well-being, identified also in previous studies (Pyhältö et al., 2009; Stubb et al., 2011; Peltonen et al., 2017). Interestingly, although medicine presents typical group-based discipline, i.e., the basic unit for conducting research is a research group providing the platform for researcher education, according to our results only about half of the candidates reported that they were engaged in a research group. This implies that formal research group structure does not automatically guarantee an experience of membership or a well-functioning collaboration with the research group.

The results showed that the PhD candidates who were working in a hospital or clinical unit had lower risk of experiencing burnout and were more likely to experience high levels of study engagement than others. This means that undertaking one’s doctoral degree when having clinical responsibilities might protect the PhD candidates from study burnout and support their study engagement. On the contrary, Kusurkar et al. (2021) found that candidates in clinical departments had lower autonomy and higher levels of conflict between work responsibilities, especially among those PhD candidates who were working with patients. A variety of factors may explain our finding. The relevance of the research itself and doctoral studies in general might become apparent in the clinical work and hence, be a source of research engagement (see also Vekkaila et al., 2013). On the other hand, the candidates engaging in clinical work might have more extensive support networks to draw from as a resource for their studying and recovery when needed. They might be also less stressed by their career prospects after completing the PhD degree or they might be aiming for a non-academic career to reduce the stress caused by the doctoral studies (see Nagy et al., 2019). In addition, financial security may explain the differences in burnout levels: Clinical PhD candidates typically receive a much higher salary than PhD candidates who do not have clinical training or employment. In addition, basic science researchers will typically rely on external, competitive funding not only for the research work itself but even for maintaining a position at the university, thereby having much lower job security than their clinically active counterparts, who always have the chance of increasing the proportion of clinical work, should funding for research be scarce.

International PhD candidates did not differ in their likelihood of belonging to any subgroup. As previous studies have suggested that although international students might be prone to experience stress (Pappa et al., 2020), they are also likely to be motivated and satisfied with their studying (Harman, 2003; Sakurai et al., 2017), and thus be likely to experience research engagement. To our knowledge, no earlier study has looked at engagement and burnout of international doctoral students specifically in the medical research education, a context that tends to be extremely international and intercultural. Based on this finding, it seems that there were no distinctive differences between the international and native PhD candidates regard to their engagement-burnout-profiles. Accordingly, this suggests that the international PhD candidates in the field of medicine are highly heterogeneous group in terms of study well-being, not primarily determined by their status as international students. For example, it might be that whether they experienced working alone or within a research group or were clinical vs. basic science medical PhD candidates, were more significant in terms of their well-being than being an international PhD student.

The PhD candidates within the profiles displaying moderate levels of engagement and moderate or high levels of burnout symptoms more often reported intention to quit the doctoral degree than those with high levels of engagement, which was in line with previous findings (Anttila et al., 2015; Cornér et al., 2017). Hence, in addition to having mental health benefits, high levels of experienced engagement are related to study progress among PhD candidates in the field of medicine. Accordingly, investing in developing engaging doctoral education environments has potentially significant individual and organizational benefits, considering that according to previous studies, drop-out rates among the PhD candidates typically range from 25 to 60% (e.g., Council of Graduate Schools, 2004; Golde, 2005; McAlpine and Norton, 2006; Gardner, 2009).

### Limitations of the Study

There are some methodological limitations in the study that need to be considered when interpreting the results. First, the criteria for selecting the number of profiles were ambiguous (Nylund et al., 2007), and hence, further studies exploring whether similar profiles can be found among other groups of PhD candidates are needed. For example, models for how profiles can be reproduced in new samples are being developed and may be helpful in exploring the well-being of PhD candidates across different medical research contexts (e.g., Gillet et al., 2021). Second, it is important to note that due to cross-sectional design, causal or process-related conclusions between study well-being and dropping out cannot be drawn. Third, the survey was sent to all doctoral students at the university simultaneously. Although the number of students who responded is sufficient for the analyses conducted, the sample only represents 36% of all doctoral students enrolled in the program. This should be kept in mind when generalizing, as we do not know whether self-selection might have affected the results. Fourth, the study was carried out in a specific social-cultural country context and in health sciences, accordingly one should be careful in drawing conclusions based on the results, across the doctoral education systems or disciplines. Last, it is important to note that data were collected before the COVID-19-pandemic. The pandemic has affected both the clinical and basic-science doctoral students in many ways. Further studies are needed to explore how stress, engagement and well-being of doctoral students working in the medical context have been affected by the pandemic at its different phases and afterwards.

## Conclusion

Undertaking a PhD in medical fields is an engaging experience for most of the PhD candidates. However, the results suggested that there are several PhD candidates with high or increased risk of burnout. Thus, it seems that individual differences occur between PhD candidates in terms of their well-being. For individuβals displaying a higher risk of burnout, it was more common to experience studying alone in their PhD compared to those with lower burnout risk. In addition, the lower risk of burnout was related to working in a clinical unit or hospital. Therefore, it can be concluded that in the field of medicine, working in research group, and in a clinical unit or hospital during their PhD can help buffering study burnout and provide sources of research engagement.

### Practical Implications

The results of the present study can be used by educational developers and staff trainers working with doctoral education. The stressors experienced by basic science PhD candidates in the highly competitive, externally funded research universities need to be taken into consideration by supervisors and policymakers. Particular attention should be paid to the candidates who experience that they are studying alone. Supervisors should be encouraged to be particularly careful in mapping out the actual support networks of their PhD candidates, instead of just formal connections to officially defined research groups. Moreover, the similarities and differences between the conditions of the clinical and non-clinical PhD candidates are worth discussing, as they work in the same general setting. The positive news for medical universities is that despite the pressures and competing responsibilities, the medical research setting is often experienced as engaging and does not automatically lead to burnout, a message worth spreading in this community engaged with cutting-edge, life-saving academic research. The study also has implications for policymakers: the findings highlight the importance of surveillance of the occupational health within the hospitals to check the psychosocial risk factors for staff undergoing research education, not merely that of residents and other health care workers.

The results also provide directions for future research on PhD candidates’ well-being. Our findings suggested that although an official membership in a research group is common in medical university, over half of the participants in this study reported that they were working alone. Working alone instead of within a research group was more common in profiles with higher burnout levels and lower levels of engagement. Therefore, reasons for the finding that most of the participants experienced working alone needs to be studied further. For example, investigation is needed to see if working alone is an active choice of a candidate or whether it represents a failure of the research education system in ensuring a supportive setting for doctoral students. In such further investigations, special attention should be paid to the actual networks, communities of practice and support. Also, factors involved in medical doctoral students’ engagement and burnout warrant closer investigation. As engagement may be more of a day-to-day experience, while burnout takes more time to develop (Sonnentag, 2017), it might be useful to look more closely at the sources of engagement for both the clinically active and the basic science subgroups of medical PhD candidates, both to identify them more precisely and to investigate the variability and trajectory of them. Given the highly competitive, high-pressure nature of research-oriented medical contexts, it might also be useful to look at experiences of exhaustion as separate from fully developed burnout, as recent research indicates that weariness does not necessarily develop into more serious burnout (Gustavsson et al., 2010; Gillet et al., 2021). For PhD candidates, supervisors, and decision-makers in these competitive environments, where high workload is more the norm than the exception, a more detailed understanding of these processes would be invaluable in terms of identifying high-risk situations and individuals in urgent need of help.

## Data Availability Statement

The raw data supporting the conclusions of this article will be made available by the authors, without undue reservation.

## Ethics Statement

The research was approved by the Swedish Central Ethical Review Board (Ref. No#2015/1626-31/5). The participants provided their written informed consent to participate in this study.

## Author Contributions

LT, KP, AB, and JN have contributed to writing the original draft and editing it. AB has contributed to data collection and project administration. LT has contributed to conducting the analyses. All authors contributed to the article and approved the submitted version.

## Conflict of Interest

The authors declare that the research was conducted in the absence of any commercial or financial relationships that could be construed as a potential conflict of interest.

## Publisher’s Note

All claims expressed in this article are solely those of the authors and do not necessarily represent those of their affiliated organizations, or those of the publisher, the editors and the reviewers. Any product that may be evaluated in this article, or claim that may be made by its manufacturer, is not guaranteed or endorsed by the publisher.

## Footnotes

^1^Time devoted to a thesis is more than 4 h/week.
