# Supplementary material for: Study Engagement and Burnout of the PhD Candidates in Medicine: A Person-Centered Approach
Source: Front Psychol. 2021 Nov 23;12:727746. doi: 10.3389/fpsyg.2021.727746 (PMC8650111; doi:10.3389/fpsyg.2021.727746)
Supplement: Supplementary file 1 [file Table_1.docx]

| Scale | Items |
| --- | --- |
| Research engagement | When I conduct my research, I feel that I am bursting with energy. |
|  | When doing my research, I feel vigorous. |
|  | I am enthusiastic about my research. |
|  | I feel happy when I start working on my research. |
|  | My research inspires me. |
| Exhaustion | I feel overwhelmed by the workload of my doctoral research. |
|  | I sleep badly because of matters related to my doctoral research. |
|  | I worry over matters related to doctoral research during my free time. |
|  | The pressure of my doctoral dissertation causes me problems in my close relationships with others. |
| Cynicism | I feel that my doctoral dissertation is useless. |
|  | I feel that I am losing interest in my doctoral research. |
|  | I have difficulties in finding any meaning to my doctoral dissertation. |
|  | I feel like I used to have higher expectations of my doctoral research than I do now. |
|  | I feel that I fail at my doctoral research. |

**Appendix 1. The scales and items**
